# Design and Evaluation of Complex Polypeptide-Loaded Dissolving Microneedles for Improving Facial Wrinkles in Different Areas

**DOI:** 10.3390/polym14214475

**Published:** 2022-10-22

**Authors:** Mengzhen Xing, Han Liu, Fanda Meng, Yuning Ma, Suohui Zhang, Yunhua Gao

**Affiliations:** 1 Key Laboratory of Photochemical Conversion and Optoelectronic Materials, Technical Institute of Physics and Chemistry, Chinese Academy of Sciences, Beijing 100190, China; 2 Key Laboratory of New Material Research Institute, Department of Acupuncture-Moxibustion and Tuina, Shandong University of Traditional Chinese Medicine, Jinan 250355, China; 3 Technical Institute of Physics and Chemistry, University of Chinese Academy of Sciences, Beijing 100049, China; 4 School of Clinical and Basic Medical Sciences, Shandong First Medical University & Shandong Academy of Medical Sciences, Jinan 250000, China; 5 Beijing CAS Microneedle Technology Ltd., Beijing 102609, China

**Keywords:** complex polypeptide, dissolving microneedles, needles height, efficacy assessments, facial wrinkles, safety evaluation

## Abstract

Wrinkles are one of the most intuitive manifestations of skin aging. Complex polypeptide-loaded dissolving microneedles (CP-DMNs) for facial wrinkles in different areas have been developed and evaluated for the first time. In optimizing formulations, we compared the differences in CP-DMNs heights on skin insertion depth and skin repair and healing. Furthermore, systemic safety experiments were carried out to provide a reference for clinical application. On this basis, an 84-day efficacy assessment based on the improvement of facial wrinkles in different areas and a comparison between CP-DMNs vs. placebo was performed on 30 healthy subjects. As a result, DMNs with a height of 300 μm presented sufficient strength to pierce the stratum corneum with minimized skin damage. In addition, CP-DMNs possessed excellent biological safety and skin compatibility for clinical application. Compared with placebo, CP-DMNs exhibited obvious improvements in wrinkles distributed in the corners of eyes, under-eyes, and nasolabial folds. Furthermore, after using CP-DMNs for 84 days, facial wrinkles in five different areas were smoothed. In short, the complex polypeptides showed apparent anti-wrinkle efficacy with the aid of DMNs technology, and CP-DMNs seemed to work better on deeper wrinkles, such as frown lines and nasolabial folds.

## 1. Introduction

As the largest organ and the first natural protective barrier of the human body, the skin protects the body from external physical, chemical substances, and microorganisms [1]. However, genetic and environmental factors affect the skin, which gradually starts to display signs of ageing [2,3]. Among them, the production of wrinkles is one of the most intuitive manifestations of skin ageing [4]. For those who pay great attention to their facial outlook, the appearance of wrinkles may cause several psychosocial issues, such as excessive anxiety and frustration with self-esteem.

Currently, the anti-ageing active substances used in cosmetics mainly include retinoids, antioxidants, polypeptides, and other functional additives [5,6,7]. It was necessary to highlight that those anti-wrinkle polypeptides have been widely applied in high-end brand skin care products, due to their high safety, significant effects, and low dosage [7]. It was known that each structural peptide has a clear and accurate skin action target based on the characteristics of peptides, and the combination of peptides with different mechanisms has been reported to enhance efficacy [8]. Nevertheless, because anti-wrinkle peptides usually have a large molecular weight (>500 Da) and strong hydrophilicity, it is difficult to penetrate the stratum corneum of the skin [9,10].

Dissolving microneedles (DMNs) are a minimally invasive transdermal delivery technology. They can be directly loaded with active ingredients and pierce the stratum corneum barrier to achieve effective drug delivery. It has been reported that dissolving microneedles loaded with polypeptides were used for wrinkles treatment, and an evident efficacy was observed [11,12]. However, most literature studies have only targeted wrinkles around the eyes [13,14]. According to their distribution areas, facial wrinkles are divided into crow’s feet, under-eye fine lines, forehead lines, frown lines, nasolabial folds, etc. [15]. Based on the unique administration of DMNs, whether there are significant differences in wrinkle improvement when they were applied to wrinkles in different areas has not been reported.

In ongoing studies, complex polypeptides consist of acetyl hexapeptide-8, palmitoyl pentapeptide-4, palmitoyl tetrapeptide-7, and oligopeptide-1, which have been used as active ingredients for wrinkle improvement. Due to their biocompatibility and skin friendliness, hyaluronic acid (HA) and polyvinylpyrrolidone (PVP) were used as the primary matrix materials of DMNs [16,17]. The safety of CP-DMNs has been comprehensively investigated to provide a reference for clinical application. Based on this, a single-blind controlled vs. placebo study was designed to evaluate the efficacy and safety of DMNs. Moreover, wrinkles in five different areas: forehead, eyebrows, corners of the eyes, under the eyes, as well as nasolabial folds were administered with DMNs for 84 days to compare the difference in wrinkle improvement in different areas.

## 2. Materials and Methods

### 2.1. Materials

Oligopeptide-1, acetyl hexapeptide-8, and palmitoyl pentapeptide-4 were purchased from Jisheng Biopharmaceutical Co., Ltd. (Sichuan, China). Hyaluronic acid (HA, Mw: 240,000) was purchased from Freda Biotechnology Co., Ltd. (Shandong, China). Polyvinylpyrrolidone (PVP, Mw: 10,000) was ordered from Boai NKY Pharmaceutical Co., Ltd. (Henan, China). Dulbecco’s modified Eagle medium (DMEM) and minimum essential medium (MEM/EBSS) were bought from Hyclone (Logan, UT, USA). Fetal bovine serum (FBS), penicillin–streptomycin (P/S), and 2.5% Trypsin (10×) were obtained from Gibco (Rockville, MD, USA). CCK-8 kit was supplied by Dojindo (Japan). The fresh rabbit blood and Tris buffer were purchased from Solarbio Tech Co., Ltd. (Beijing, China). Lecithin Tween-80 Nutrient Agar (LTNA) and Rose Bengal Agar (RBA) medium were purchased from Qingdao Hope Bio-Technology Co., Ltd. (Shandong, China). Other chemicals were chromatographic grade.

Ex vivo porcine cadaver skin was supported by Kaikai Tech Co., Ltd. (Shanghai, China). Fibroblasts (L929) and human immortalized epidermal cells (HaCaT) were kindly given as a present by Professor Zhongwei Niu, Technical Institute of Physics and Chemistry (Beijing, China). Sprague Dawley (SD) rats (male, 8 weeks old, 220 ± 20 g) and New Zealand white rabbits (half male and half female, 2.0 ± 0.5 kg) were obtained from SPF Biotech Co., Ltd. (Beijing, China). Procedures for animal studies were approved by the Institutional Animal Care and Utilisation Committee of the Technical Institute of Physics and Chemistry, CAS (approval number, LHDW-210116; 23 November 2021, and IACUC-IPC-21039; 16 May 2021). The animal experiments followed the Guide for the Care and Use of Laboratory Animals (Eighth Edition, 2011).

### 2.2. Volunteers

According to specific inclusion and non-inclusion criteria, as shown in Table S1, 30 (33 included) healthy female subjects aged 40–70 years with visible facial wrinkles were enrolled by a board-certified dermatologist. The study was carried out under dermatological control—in compliance with the ethical principle for medical research (Helsinki declaration and amendments). Additionally, the experiments were validated by the medical ethic committee of Xiyuan Hospital, China Academy of Chinese Medical Sciences (approval number: 2021XL006-4).

### 2.3. Preparation of CP-DMNs

To prepare CP-DMNs, the compounds consisting of oligopeptide-1 (0.15%, *w*/*v*), acetyl hexapeptide-8 (0.03%, *w*/*v*), and palmitoyl pentapeptide-4 (0.03%, *w*/*v*) were dissolved in ultrapure water to acquire an active ingredient solution. Afterwards, 7% (*w*/*v*) HA and 3% (*w*/*v*) PVP were performed as matrix materials due to their excellent needle-forming and skin compatibility [17,18]. The DMNs solution was made by mixing the active ingredient solution and matrix solution. Subsequently, the polydimethylsiloxane (PDMS) mold was used to develop the CP-DMNs in batches, as described in our previous method [19]. First, the laser micromachining technology was applied to prepare the microneedles master, made of stainless steel with sharp tips. Secondly, the inverse PDMS mold was fabricated by casting the dimethyl siloxane and initiator mixture onto the microneedle master structure. Next, a micro-molding technology was performed through a four-step procedure, including (i) DMNs solution was quantitatively transferred to the PDMS mold via a pipette gun; (ii) PDMS mold was vacuumed to make micropores filled with DMNs solution; (iii) MNs solution was dried at ambient temperature with a relative humidity of 20–30% for 2 h; and (iv) CP-DMNs were acquired by removing them from PDMS mold.

### 2.4. Characterizations of CP-DMNs

First, PDMS molds with needle heights of 230 μm, 500 μm, and 700 μm were used to prepare microneedles of different heights. The specific preparation method is described in Section 2.3. The actual heights were measured via a fluorescence microscope (BX51, OLYMPUS, Japan) and a stereo microscope (SMP1000, Nikon, Japan). Then, the skin insertion experiments were performed on three kinds of DMNs. Then, the DMNs were removed, and the skins were immersed in 4% paraformaldehyde for 4 h. The fixed tissues were embedded in paraffin and sectioned at a thickness of 4 μm. After staining with hematoxylin and eosin (H&E), the paraffin sections were imaged under a Pannoramic 250 Flash System (3DHISTECH, Hungary).

For evaluating the skin repair capability after the application of DMNs with different needle heights, DMNs loaded with 4 mg/mL Trypan blue dye were prepared using PDMS molds with needle heights of 230 μm, 500 μm, and 700 μm, respectively. Three DMNs were inserted into the rats’ skin with the help of a homemade applicator (20 N/cm^2^) for 20 s. After removing DMNs for 0, 10, 30, 60, and 180 min, pictures were taken to record the pinhole array on the rats’ skin to study the skin’s self-healing ability.

To investigate the intradermal dissolving properties of CP-DMNs, the ex vivo suckling pig skin was used as the model skin. Briefly, the hydrogel backing was attached to DMNs’ backing layer to facilitate the fixation of microneedles on the skin. Then, CP-DMNs were applied to the skin with the help of a homemade applicator (20 N/cm^2^) for 20 s, and maintained for 0, 10, and 30 min, respectively. Afterwards, the residual microneedles were placed under the fluorescence microscope (BX51, Olympus, Japan) to measure the height changes of CP-DMNs compared with the initial CP-DMNs.

### 2.5. In Vitro Safety Studies of CP-DMNs

Cell viability was measured using a CCK-8 kit [18]. Firstly, six pieces of CP-DMNs were sterilized via exposure to UV overnight and immersed into 3 mL of DMEM and MEM/EBSS medium for 72 h (each medium containing three pieces of CP-DMNs). Then, the supernatants were collected and diluted into 100%, 50%, and 25% with DMEM or MEM/EBSS medium. Subsequently, 10% FBS was added to each solution to acquire a series of DMN-extracted solutions with different concentrations. Simultaneously, fibroblasts (L929) and human-immortalized epidermal cells (HaCaT) were seeded into 96-well plates cultured (5 × 10^3^/well) with DMEM and MEM/EBSS mediums, respectively. After incubation at a 37 °C, 5% CO_2_ incubator for 24 h, different concentrations of CP-DMN extracted solutions were added to the corresponding wells and continued to culture for another 24 h. Meanwhile, the normal medium and 5% dimethyl sulfoxide (DMSO) were performed as the control group and positive group, respectively. After being cultured for 24 h, 10% CCK-8 was added into each well for 2 h. The absorbance of cell culture at 450 nm was determined with a microplate reader (RT-2100C, Rayto, China). The cell viability was obtained by the following formula:

Cell viability = (A_sample_/A_control_) × 100% (1)

A_sample_ and A_control_ represent the absorbance of the sample and control groups, respectively.

Consistent with the sample preparation method used for cytotoxicity testing, Tris buffer (3 mL) was chosen as the solvent to dissolve three pieces of CP-DMNs for 24 h. Then, the supernatants were diluted into different concentrations of 100%, 50%, and 25%, respectively. Fresh rabbit blood was mixed with 4 mL Tris buffer and centrifuged at 1000× *g* for 10 min to acquire the erythrocyte. Afterwards, the erythrocyte was washed with Tris buffer for three times until the supernatant was colorless. The erythrocyte suspension (5%) and CP-DMN-extracted solutions with different concentrations were, respectively, mixed in centrifuge tubes and incubated at 37 °C for 1 h. After centrifuging the mixture with 1000× *g* for 10 min, the supernatant of each group was transferred to a 96-well microplate. The microplate reader (RT-2100C, Rayto, China) was used to measure the absorbance at 570 nm. Moreover, 0.1% Triton-X and Tris buffer were performed as the positive control and negative control, respectively [20]. The hemolysis radio was calculated according to the following formula:

Hemolysis radio = (H − Hn)/(Hp − Hn) × 100%(2)

H, H_n_ and H_p_ stand for the absorbance of sample, negative, and positive control groups, respectively.

Microbial limit tests of CP-DMNs were established to ensure the safe application according to Safety and Technical Standards for Cosmetics (2015). First, LTNA and RBA mediums were prepared according to the manufacturer’s protocol. After autoclaving, they were placed in a constant temperature water bath at approximately 55 °C. The CP-DMNs produced in a class 10,000 clean room were soaked in 0.9% sodium chloride injection and shaken to obtain a test solution of 0.1 g/mL. Add 1 mL of the test solution into a sterile Petri dish, then repeat four times to acquire 4 parallel Petri dishes. Then, 15–20 mL of LTNA medium was poured into two dishes, and the same volume of RBA medium was added to the other two dishes. After the agar was naturally cooled and solidified, the LTNA plates were placed in an incubator at 36 ± 1 °C for 48 ± 2 h to check the total number of bacteria. Moreover, the RBA plates were transferred to an incubator at 28 ± 2 °C for 5 days, and the amount of mold and yeast in the plates was counted after cultivation.

### 2.6. In Vivo Skin Irritation Study of CP-DMNs

The skin irritation assessment was performed based on our previous report [21]. The New Zealand white rabbits (half male and half female, 2.0 ± 0.5 kg) were housed in an ordinary condition and fed a regular diet for a week to adapt to the environment. The dorsal hair was removed to facilitate the use of DMN patches, and the skin was divided into four sections, namely: part 1, part 2, part 3, and part 4. Parts 1 and 4 were used as the experiment group, where they were applied with CP-DMNs. Meanwhile, parts 2 and 3 were attached with blank DMNs. After using it for 10 h, all DMN patches were removed. Observe and record the skin irritation conditions, such as erythema and edema.

### 2.7. Clinical Studies of CP-DMNs

A split-face randomized controlled pilot study and a self-comparative test of facial wrinkles in different areas were simultaneously performed on thirty subjects. In detail, subjects were required to apply CP-DMNs on the right or left crow’s feet, under-eye fine lines and nasolabial folds (according to a previously defined randomization list) and the placebo (blank DMNs) was used on the control lateral wrinkles. Moreover, all subjects were told to use CP-DMNs patches on their forehead and frown lines. The detailed DMNs distribution diagram was shown in Figure S1. Then, DMN patches were applied to the corresponding wrinkles once a day before going to sleep and kept on all night (at least continuative 6 h). On the next day, all DMNs patches were removed, and the test lasted for 84 days. The efficacy was measured at baseline (T0) and after 14 (T14), 28 (T28), 56 (T56) and 84 (T84) days of product application. Monitored parameters including a wrinkles profilometry with Primos CR (GF Messtechnik GmbH, Teltow, Germany), standard imaging based on VISIA-CR (Canfield, OH, USA), safety evaluation, and self-assessment according to the questionnaire. All subjects were provided base creams without any cosmetic claim during the study period to standardize the test condition.

### 2.8. Statistical Analysis

All quantified data were analyzed by IBM SPSS Statistics 23.0 (IBM SPSS Inc., Chicago, IL, USA). Whether it was a comparison within a group or a comparison between test groups and control groups, the paired t-test was used for statistical analysis, when the data were normally distributed. Otherwise, the rank sum test was performed. The significant threshold of *p* < 0.05, *p* < 0.01, *p* < 0.001 are marked with *, ** and ***, respectively.

## 3. Results and Discussion

### 3.1. Preparation and Characterization of DMNs

**Figure 1:**
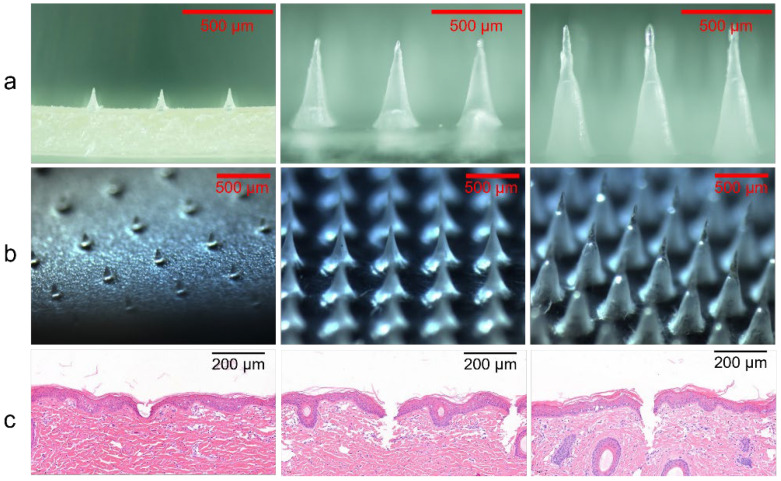
Images of needles morphology (**a**), arrays (**b**), and intradermal penetration depth (**c**) of DMNs with needle heights of 230 μm, 500 μm, and 700 μm, obtained by fluorescence microscope, stereo microscope, and tissue slices, respectively.

**Figure 2:**
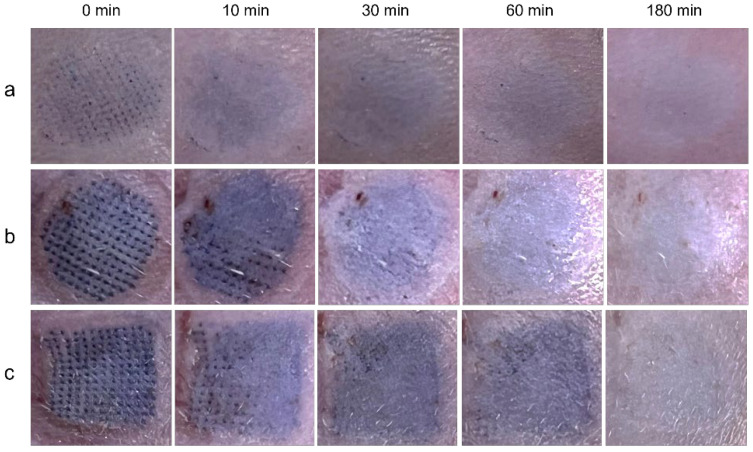
The self-repair time of pinhole array on rats’ skin after DMNs with different needle lengths were inserted (**a**): 230 μm; (**b**): 500 μm; (**c**): 700 μm.

The actual heights of DMNs prepared with different female moldings were 227 ± 6 μm, 498 ± 4 μm, and 707 ± 10 μm as shown in Figure 1a,b. With sharp needle tips, it was easy for DMNs to destroy the stratum corneum barrier. Normal skin consists of three layers: epidermis, dermis, and subcutis [22]. Moreover, the epidermis is located at approximately 50–100 μm under the outer skin, which is the controlled release layer for the percutaneous absorption of drugs [23]. Additionally, due to the elasticity of the skin and the cushioning effect of subcutaneous fat, the actual penetration depth was usually shorter than the height of DMNs [24,25]. The results of histological analysis displayed that insertion depths of 230 μm DMNs, 500 μm DMNs, and 700 μm DMNs were 85 ± 12 μm, 182 ± 18 μm, and 246 ± 10 μm, respectively, under the force of 20 N/cm^2^ (Figure 1c). Thereby, 230 μm DMNs presented sufficient strength to pierce the stratum corneum and minimize damage to the skin. In the in vivo skin repair test, the array of 230 μm-DMNs group disappeared within 30 min (Figure 2a), indicating that the damaged epidermis has been quickly healed through self-repair. In contrast, 500 μm DMNs and 700 μm DMNs groups were repaired for 60–180 min (Figure 2b,c). As we expected, on account of a shallower insertion depth and a lighter local micro-injury, 230 μm DMNs exhibited the fastest healing speed. A faster healing efficiency reduces the possibility of wound infection, so it has higher skin safety and patient compliance.

**Figure 3:**
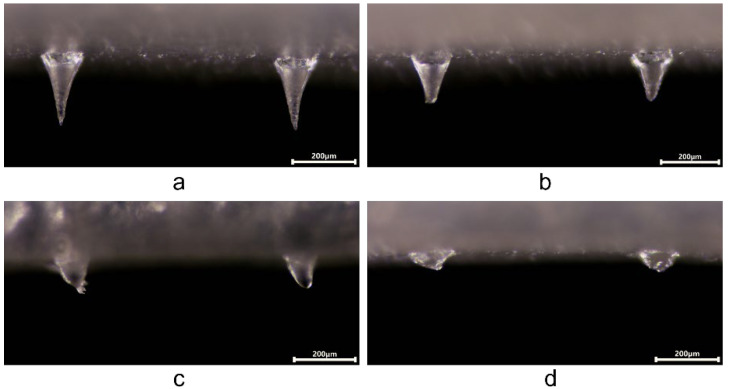
Microscopy images of morphological changes in CP-DMNs before (**a**) and after insertion into ex vivo suckling pig skin for 20 s, and maintained for 0 (**b**), 10 (**c**), 30 (**d**) min (scale bar is 200 μm).

In addition, the transdermal dissolution study demonstrated that the tips of CP-DMNs quickly dissolved 20 s after being inserted into the skin (Figure 3b). After being applied for 10 min, the residual needles were less than half of the initial microneedles (Figure 3a,c). Finally, the needles had wholly entered the isolated skin after being maintained for 30 min (Figure 3d). The rapid dissolution of CP-DMNs could be attributed to the instant solubility properties of the matrix materials (HA and PVP), which facilitated the effective entry of the active ingredients into the dermis layer. As for the base layer of CP-DMNs, they were dissolved through the penetration capability of microchannels and skin hydration, thereby assisting the drug in continuing to spread into the skin. 

### 3.2. In Vitro and In Vivo Safety Studies of CP-DMNs

**Figure 4:**
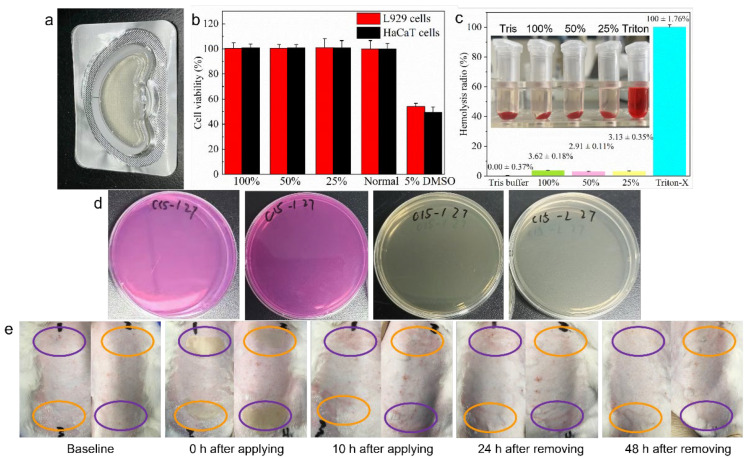
A systemic safety evaluation of complex polypeptide-loaded dissolving microneedles. The packaged CP-DMNs with sterilized blister and aluminum–plastic bag (**a**). The cell viability results of CP-DMNs for L929 and HaCaT cells (**b**). The results of erythrocyte hemolysis for CP-DMNs (**c**). The LTNA and RBA plates after being incubated to check the existence of bacteria, mold and yeast in CP-DMNs (**d**). The in vivo skin irritation research of CP-DMNs on rabbits’ skin (**e**).

Before designing the human efficacy assessment, we verified the safety of CP-DMNs through a series of studies such as cytotoxicity, erythrocyte hemolysis, microbial limit test, and skin irritation. The entire production process of DMNs was carried out in a class 10,000 clean room. The sterilized blisters and aluminum–plastic bags were used for packaging to ensure the safety and effectiveness of the product (Figure 4a). The cytotoxicity experiment displayed no significant difference in cell viability between the DMNs group and the normal medium group (*p* > 0.05). A similar result was observed in the erythrocyte hemolysis test, revealing that CP-DMNs have excellent cytocompatibility. On the contrary, the positive control group showed a decrease in cell viability and obvious hemolytic reaction (Figure 4b,c). The biological safety of CP-DMNs is a vital prerequisite to ensuring the safe use of products. The results of a microbial limit test showed that neither bacteria, mold, nor yeast were detected in the DMNs extract (Figure 4d). In the in vivo skin irritation research, slight erythema appeared in the applied areas after peeling off patches, both in CP-DMNs and blank DMNs groups. After 24 h, the erythema gradually subsided due to the self-healing ability of the skin. There was no significant difference between CP-DMNs and blank DMNs groups, indicating that adding complex polypeptides would not induce any adverse skin reactions. Finally, the erythema completely disappeared, and the skin returned to its initial stage within 48 h (Figure 4e). Based on these studies, it was concluded that CP-DMNs possessed great safety and could be used for human efficacy assessment.

### 3.3. Clinical Studies of CP-DMNs vs. Placebo

**Figure 5:**
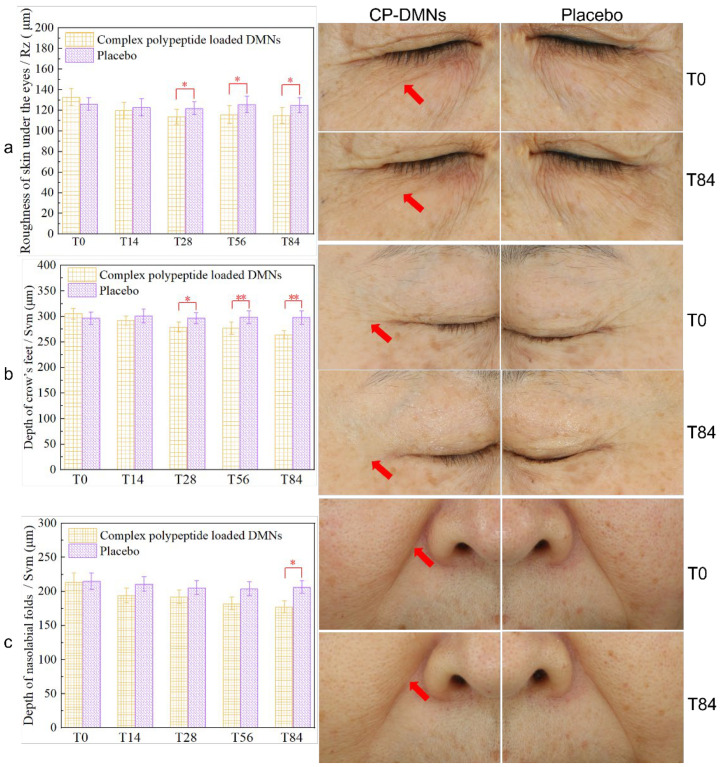
The results of the wrinkles profilometry and standard imaging based on under-eye fine lines (**a**), crow’s feet (**b**), and nasolabial folds (**c**) between CP-DMNs and placebo (blank DMNs). The significant threshold of *p* < 0.05 and *p* < 0.01 are marked with * and **, respectively.

A split-face randomized controlled pilot study was designed to compare the efficacy between CP-DMNs and blank DMNs on under-eye fine lines, crow’s feet, and nasolabial folds, respectively. The results are presented in Figure 5. Unlike under-eye fine lines, the appearance of crow’s feet and nasolabial folds are mainly associated with long-term repeated facial expressions, such as smiles. As a result, they are also called expression lines. Due to years of accumulation, coupled with the loss of intradermal collagen and elastin caused by aging and ultraviolet radiation, skin elasticity decreases [26,27,28]. Eventually, the dynamic expression lines gradually turn into static lines. They are usually rougher and deeper than fine lines (for instance, under the eye wrinkles) formed on local dry skin. Therefore, the measurement parameter of under-eye fine lines was skin roughness (Rz), and the evaluation index of the crow’s feet as well as nasolabial folds are the depth of skin wrinkles (Svm). For under the eye wrinkles and crow’s feet, a difference between CP-DMNs group and placebo was observed after being used for 28 days (*p* < 0.05), as shown in Figure 5a,b. Moreover, there was a significant difference in the crow’s feet group after 56 days of continuous application (*p* < 0.001) (Figure 5b). Additionally, 84 days later, compared to the placebo group, a noticeable improvement in nasolabial folds was found in the CP-DMN groups (*p* < 0.05) (Figure 5c). More intuitively, standard images of baseline and 84-day treatment with CP-DMNs or placebo on the subjects’ crow’s feet, under-eye fine lines, and nasolabial folds were taken through VISIA-CR. As indicated by the red arrow, the application of the CP-DMNs acquired a visible improvement in the three types of wrinkles, while that of placebo was limited. In fact, individual evident improvements were observed in the placebo group, which can be attributed to the moisturizing effect of HA (matrix materials of DMNs). Nevertheless, the total anti-wrinkle results were weaker than the CP-DMNs group. A formulation composed of arginine/lysine polypeptide, acetyl octapeptide-3, palmitoyl tripeptide-5, adenosine, and seaweed extracts was used in the monocentric 12-week clinical trial. Furthermore, a multi-targeted effect of the combination was found, and the synergistic efficacy of wrinkles improvement between ingredients with different mechanisms was concluded [11]. Similarly, in our formulations, complex polypeptides consisting of acetyl hexapeptide-8, palmitoyl pentapeptide-4, palmitoyl tetrapeptide-7, and oligopeptide-1 were applied based on their anti-wrinkle mechanisms [29,30,31]. After being inserted into the skin, the active polypeptides were quickly released. Pentapeptide-4, palmitoyl tetrapeptide-7, and oligopeptide-1 promoted the synthesis of collagen and elastin in the dermis and reversed the skin ageing process through reconstruction from inside to outside, thereby increasing the thickness of the dermis and improving wrinkles. For wrinkles caused by inevitable facial expressions, acetyl hexapeptide-8 can locally block nerves from transmitting contraction information, so that facial muscle contraction was weakened, thereby preventing the generation of new wrinkles.

### 3.4. Clinical Studies of CP-DMNs Applied at Different Areas

**Figure 6:**
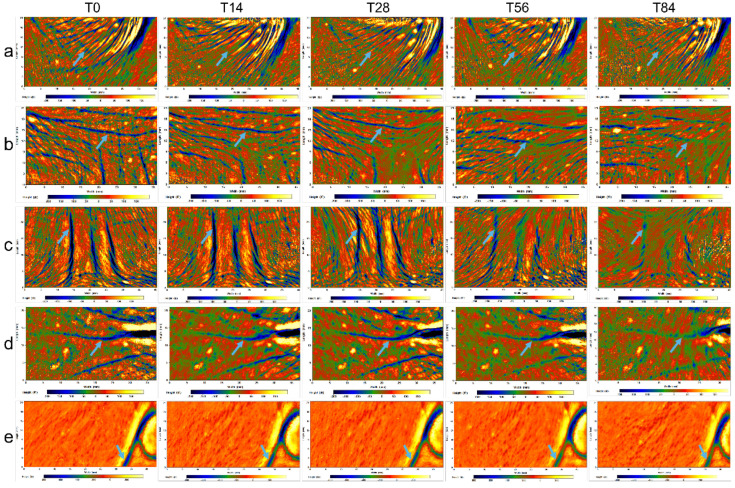
The color-coded 3D images of under-eye fine lines (**a**), forehead lines (**b**), eyebrow lines (**c**), crow’s feet (**d**), and nasolabial folds (**e**) with continuous administration for 84 days.

**Figure 7:**
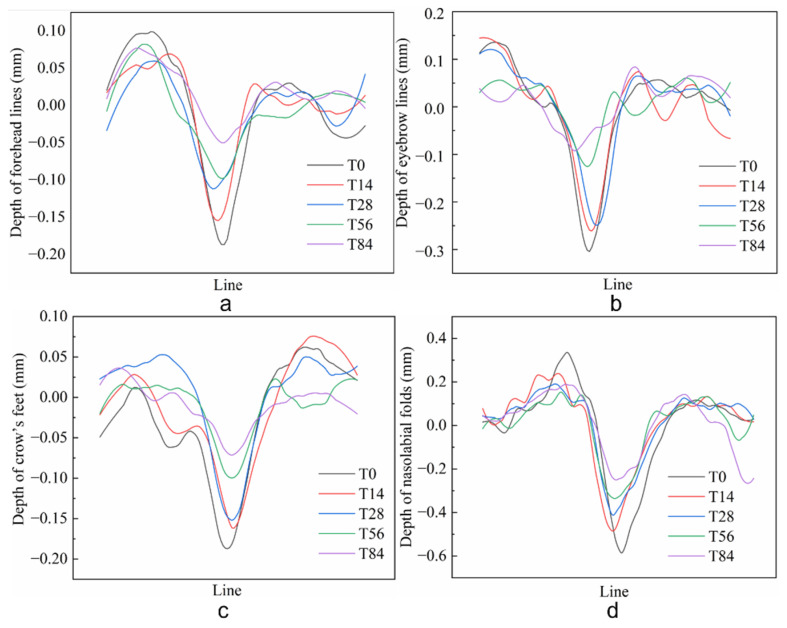
The topical line analysis images of forehead lines (**a**), eyebrow lines (**b**), crow’s feet (**c**), and nasolabial folds (**d**) with continuous administration for 84 days.

**Figure 8:**
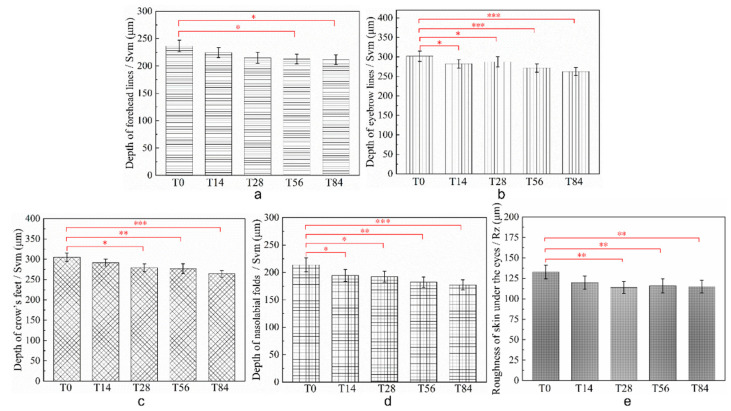
After 84 days of continuous treatment with CP-DMNs in different test areas: forehead lines (**a**); eyebrow lines (**b**); crow’s feet (**c**); nasolabial folds (**d**); and under-eye fine lines (**e**); the subjects’ wrinkle depth or skin roughness at a specific time point. The significant threshold of *p* < 0.05, *p* < 0.01, *p* < 0.001 are marked with *, ** and ***, respectively.

Like crow’s feet and nasolabial folds, forehead lines and eyebrow lines also belong to expression lines induced by repeated staring, frowning, and other expression. Compared with the fine and diffuse distribution of under-eye fine lines classified as gravity wrinkles caused by skin and muscle relaxation, the four dynamic wrinkles are clearer, deeper, and more intuitive. In Figure 6 and Figure 7, the color-coded 3D images and topical line analysis were performed to further assess the visible wrinkles resistance of CP-DMNs in five different facial areas. On the baseline, fine lines under the eyes were radially distributed, with shallow lines but a wider area. In contrast, the forehead, eyebrows, corners of the eyes, and nasolabial space were covered with clear and deep wrinkles. After using CP-DMNs for 28 days, visual improvements were observed in all the tested areas. In addition, with the extension of application time, wrinkles in five areas were gradually smoothing, and the volume of wrinkles was significantly reduced over the 84 days. Especially, both eyebrow lines and nasolabial folds presented extreme improvements as shown in Figure 6c,e as well as Figure 7b,d. Meanwhile, the overall efficacy results following the 84-day’s continuous administration were exhibited in Figure 8. Compared with the baseline, the nasolabial folds were first applied to an area that showed a significant improvement within 14 days (Figure 8d), followed by under-eye fine lines (Figure 8e), eyebrow lines (Figure 8b), and crow’s feet (Figure 8c), from which an obvious anti-wrinkle effect also emerged after application for 28 days. However, the difference appeared for forehead lines after 56 days, and the degree of improvement did not expand further at 84 days (*p* < 0.05), as shown in Figure 8a. This may be related to the fact that many subjects’ forehead lines were not apparent in the resting state, which was based on our analysis of all subjects’ standard photographs. In contrast, nasolabial folds, frown lines, and crow’s feet were all significantly improved after 84 days of treatment with CP-DMNs (*p* < 0.001). Currently, multitarget-based peptide combinations are widely used in well-known cosmetics brands. However, most prescriptions are formulated as emulsions or creams and administered by topical application [32,33]. Limited by the molecular weight and strong hydrophilicity of peptides, the effective utilization of drugs is extremely low. Nevertheless, the development of dissolving microneedles has completely solved this problem. Active ingredients can effectively enter the skin to achieve local or systemic administration by penetrating the stratum corneum. In summary, complex polypeptide-loaded dissolving microneedles are a practical approach to improving facial wrinkles, especially for deep lines. The effect was more significant. 

After 84 days of continuous use, a questionnaire survey was conducted to reflect the subjects’ use experience. During the trial, no one reported any adverse reactions or skin allergy events (such as redness, dryness, burn, sting, or itch responses). This indicated that CP-DMNs were extremely well tolerated and can be used daily. Additionally, most users thought that the independent packaging design of complex polypeptides loaded DMNs was easy to carry and convenient to use, and the DMNs patches showed a good skin fit, which is suitable for facial wrinkles in different areas. In short, 93% of subjects rated the overall preference of this product at 8 points or more (10 points is a perfect score).

## 4. Conclusions

The CP-DMNs showed excellent skin compatibility and tolerance, ensuring the safety of the clinical application. In efficacy evaluations, the complex polypeptides showed a noticeable anti-wrinkle effect with the aid of DMN technology. Moreover, the DMNs administration was a promising approach to transdermal drug delivery, showing unique advantages in skin disease treatment. The CP-DMNs worked better on deeper wrinkles, such as frown lines and nasolabial folds. Based upon this, we speculate that multiple combinations of the complex polypeptides may be used in different types of wrinkles. Further research is planned to provide an effective method for improving facial wrinkles in different areas.

## Data Availability

Data available on request due to restrictions, e.g., privacy or ethical, the data presented in this study are available on request from the corresponding author.

## Supplementary Materials

The following supporting information can be downloaded at: https://www.mdpi.com/article/10.3390/polym14214475/s1, Figure S1: The detailed DMNs distribution diagram in the subjects’ face; Table S1: Recruitment criteria for subjects: specific principles of inclusion and exclusion.

## Abbreviations

DMNs, dissolving microneedles; CP-DMNs, complex polypeptide-loaded dissolving microneedles; HA, hyaluronic acid; PVP, polyvinylpyrrolidone; DMEM, Dulbecco’s modified Eagle medium; MEM/EBSS, minimum essential medium; FBS, fetal bovine serum; P/S, penicillin–streptomycin; LTNA, Lecithin Tween-80 Nutrient Agar; RBA, Rose Bengal Agar; L929, fibroblasts cells; HaCaT, human immortalized epidermal cells; SD, Sprague Dawley rats; PDMS, polydimethylsiloxane; H&E, hematoxylin and eosin; DMSO, dimethyl sulfoxide; Rz, skin roughness; Svm, depth of skin wrinkles.
