# Supplementary material for: Design and Evaluation of Complex Polypeptide-Loaded Dissolving Microneedles for Improving Facial Wrinkles in Different Areas
Source: Polymers (Basel). 2022 Oct 22;14(21):4475. doi: 10.3390/polym14214475 (PMC9653557; doi:10.3390/polym14214475)
Supplement: Supplementary file 1 [file polymers-14-04475-s001.zip › polymers-1942827-supplementary.pdf]

Table S1. The specific inclusion and non-inclusion criteria for selecting subjects.

Inclusion criteria	Non-inclusion criteria
Female healthy subjects	Pregnant and breastfeeding woman
Age range: 40 to 70 years old	A history of allergies or sensitivity to cosmetic products, toiletries, sunscreens and/or topical drugs.
Type: Asian	Volunteers with dermatological / ophthalmological problems on the test area.
Showing visible Crow's Feet, Under the eyes fine lines, Nasolabial Folds and forehead and frown lines.	Volunteers with pharmacological treatment (locally or systemically) in progress.
Volunteers who have not involved in any other similar studies since less than two months.	Sunburn, suntan, scars, or active dermal lesions on the test area.
Willingness to not expose to sun/solar lamps during the study period.	Subjects accustomed to use tanning beds.
Willingness to not vary the normal daily routine.	Subject having a skin condition that the investigator deems inappropriate for participation.
Volunteers aware of the test procedure and having signed an informed consent form.	Subject under pharmacological treatment (locally or systemically) that could interfere with the study treatment.

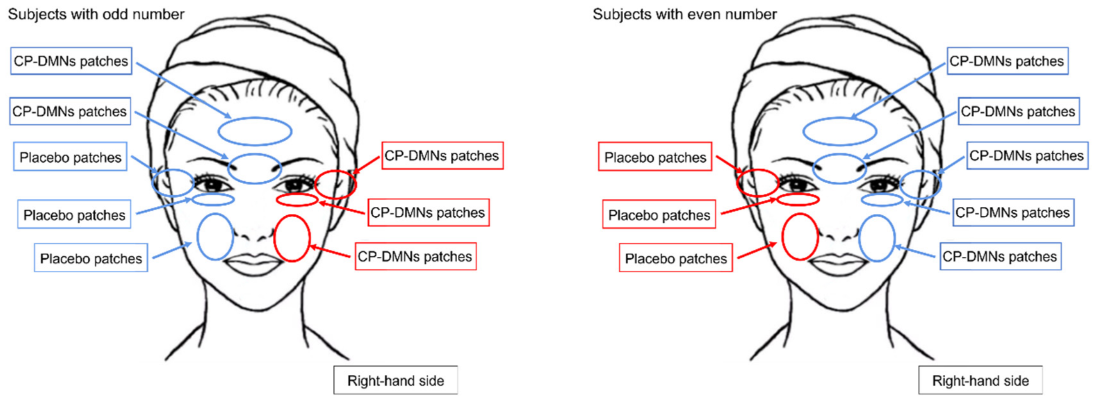

Figure S1. The detailed DMNs distribution diagram.
